# Silencing Notch4 promotes tumorigenesis and inhibits metastasis of triple-negative breast cancer via Nanog and Cdc42

**DOI:** 10.1038/s41420-023-01450-w

**Published:** 2023-05-06

**Authors:** Yuan Tian, Peipei Zhang, Yajun Mou, Wenxiu Yang, Junhong Zhang, Qing Li, Xiaowei Dou

**Affiliations:** 1 Department of Pathology, The Affiliated Hospital of Guizhou Medical University, 550004 Guiyang, Guizhou China; 2 Clinical Research Center, The Affiliated Hospital of Guizhou Medical University, 550004 Guiyang, Guizhou China; 3 Department of Orthopedics, The Affiliated Hospital of Guizhou Medical University, 550004 Guiyang, Guizhou China

**Keywords:** Breast cancer, Drug development

## Abstract

Elucidation of individual Notch protein biology in specific cancer is crucial to develop safe, effective, and tumor-selective Notch-targeting therapeutic reagents for clinical use [1]. Here, we explored the Notch4 function in triple-negative breast cancer (TNBC). We found that silencing Notch4 enhanced tumorigenic ability in TNBC cells via upregulating Nanog expression, a pluripotency factor of embryonic stem cells. Intriguingly, silencing Notch4 in TNBC cells suppressed metastasis via downregulating Cdc42 expression, a key molecular for cell polarity formation. Notably, downregulation of Cdc42 expression affected Vimentin distribution, but not Vimentin expression to inhibit EMT shift. Collectively, our results show that silencing Notch4 enhances tumorigenesis and inhibits metastasis in TNBC, indicating that targeting Notch4 may not be a potential strategy for drug discovery in TNBC.

## Introduction

Notch signal is implicated in several cancers, and aberrant activation is linked to cancer initiation, metastasis, and resistance to cancer therapy [1]. Mounting evidence pointed out that individual Notch proteins had distinct activities and outcomes in the subtype of breast cancer. For example, while Notch3 signaling exhibits the oncogenic potential in a murine breast cancer model and promotes luminal breast cancer metastasis [2–4], overexpression of Notch3 inhibits proliferation and metastasis in TNBC and is associated with better survival in TNBC patients [5–11]. However, other researchers argued that the Notch3 signal pathway promoted the growth and metastasis of TNBC [12, 13]. In TNBC cells, Notch4 maintained cancer stemness and promoted metastasis, indicating that Notch4 could be a potential therapeutic target for TNBC treatment [14, 15]. In ER^+^ breast cancer, Notch4 increases breast cancer stem cell activation and endocrine therapy resistance, proposing that Notch4 inhibition will overcome endocrine therapy resistance and decrease recurrence in ER^+^ breast cancer [16, 17].

Elucidation of the activities and outcomes of individual Notch in breast cancer subtypes aid in developing a novel therapeutic strategy that targets Notch-driven cancers with efficacy and diminishes adverse events caused by nonspecific inhibition of Notch signal [1, 18]. For example, γ- Secretase inhibitor (GSI) PF-03084014, a pan-Notch inhibitor, was reported to have no significant clinical efficacy in phase I clinical trials in patients with breast cancer. Although GSIs are ineffective as a monotherapy, the preclinical ability of GSIs to reduce cancer stem cell proliferative capacity and curtail chemoresistance has led to an investigation of clinical combinatorial strategies [19]. But treatment-related adverse events, including neutropenia, fatigue, nausea, leukopenia, diarrhea, alopecia, anemia, and vomiting, were reported in almost all breast cancer patients in PF-03084014 clinical trials [20–22]. Similar low efficacy and adverse events were reported in the other pan-Notch inhibitor RO4929097 clinical trials with breast cancer patients [23–26]. These studies showed that pan-Notch inhibitors failed to prove clinical benefits and led to adverse events in clinical trials with breast cancer. However, a phase I clinical trial of PF-06650808, a Notch3-specific antibody–drug conjugate, has demonstrated a reasonable safety profile and early efficacy signs of antitumor activity in patients with advanced breast cancer [27].

In this study, we report that the knockdown of Notch4 in TNBC cells promotes tumorigenesis and inhibits metastasis. Mechanistically, the knockdown of Notch4 upregulated Nanog, a transcription factor required for maintaining the pluripotency of embryonic stem cells [28], via Hes3 to promote tumorigenesis. Unexpectedly, the knockdown of Notch4 inhibits metastasis through downregulation of Cdc42 expression, which rendered diffuse distribution around the nucleus of Vimentin location, but not the reduction of Vimentin expression to suppress EMT shift. Hence, our demonstration showed that silencing Notch4 in TNBC cells promotes tumorigenesis and inhibits metastasis, indicating targeting Notch4 possibly not being a potential therapeutic strategy for TNBC.

## Results

### Low Notch4 expression seemingly predicts the recurrence in TNBC patients with lymph node metastasis

**Fig. 1:**
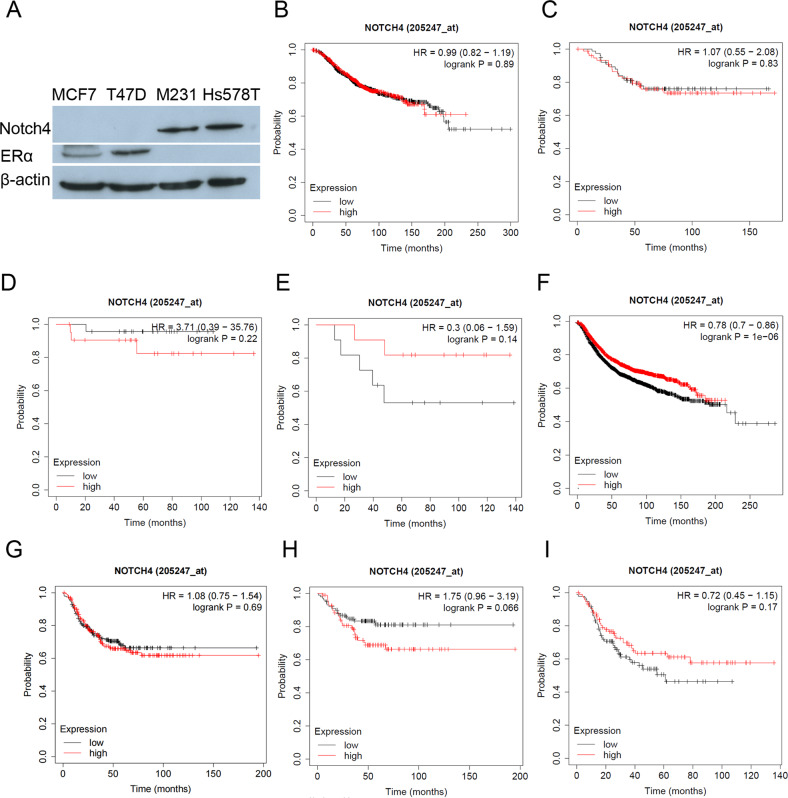
Role of Notch4 in TNBC. **A** Western blot analysis showed that Notch4 was dominantly expressed in TNBC cells. **B**, **C**, **F**, **G** OS/RFS in breast cancer and TNBC patients according to Notch4 expression in Kaplan–Meier plotter database. **D**, **H** OS/RFS in breast cancer and TNBC patients without lymph node metastasis according to Notch4 expression in Kaplan–Meier plotter database. **E**, **I** OS/RFS in breast cancer and TNBC patients with lymph node metastasis according to Notch4 expression in Kaplan–Meier plotter database.

Based on previous research and our results, we found that Notch4 was expressed predominantly in TNBC cell lines MDA-MB-231 and Hs578T but not in luminal breast cancer cell lines MCF-7 and T47D (Fig. 1A) [6, 14]. To investigate the potential role of Notch4 in TNBC, we evaluated the prognostic value of Notch4 mRNA in a large online gene chip database of breast tumors from 1880 patients for overall survival (OS) and 4934 patients for recurrence-free survival (RFS) in Kaplan–Meier plotter. We found that there is almost no correlation between Notch4 expression and OS/RFS in breast cancer and TNBC patients (Fig. 1B, C, F, G). Although higher expression of Notch4 mRNA is seemingly associated with poorer OS and RFS in breast cancer and TNBC patients without lymph node metastasis (Fig. 1D, H), lower expression of Notch4 mRNA is seemingly correlated with poorer OS and RFS in breast cancer and TNBC patients with lymph node metastasis (Fig. 1E, I). These results seemingly suggested that lower expression of Notch4 is associated with breast cancer recurrence in TNBC with lymph node metastasis.

### Silencing Notch4 promotes tumorigenesis in TNBC cells

**Fig. 2:**
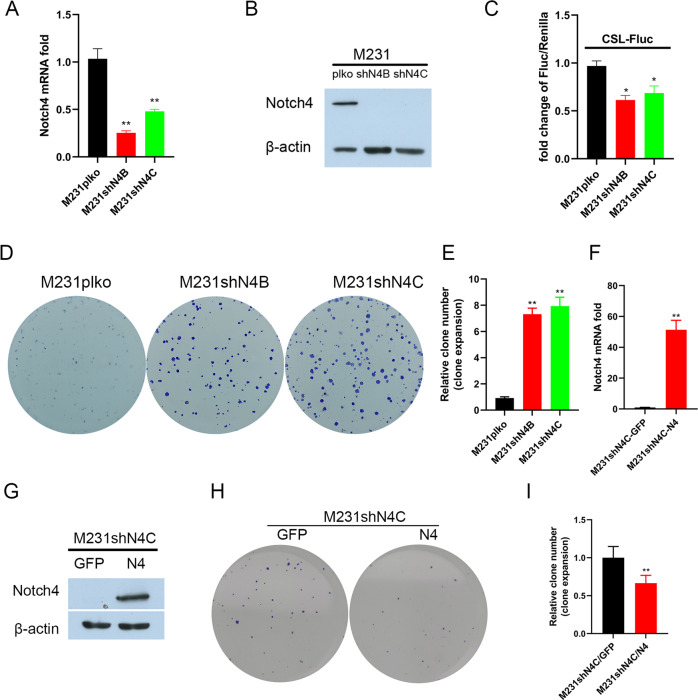
The knockdown of Notch4 of TNBC cells promotes tumorigenesis in vitro. **A** The knockdown of Notch4 in MDA-MB-231 cells was confirmed by real-time PCR. **P < 0.01. **B** Notch4 knockdown in MDA-MB-231 cells was confirmed by western blot analysis. **C** The knockdown of Notch4 in MDA-MB-231 cells was confirmed by promoter reporter assay. *P < 0.05. **D**, **E** Notch4 knockdown in MDA-MB-231 cells increased clone formation. **P < 0.01. **F** Overexpressing Notch4 in M231shN4 cells by real-time PCR. *P < 0.05. **G** Overexpressing Notch4 in M231shN4 cells by western blot analysis. **H**, **I** Overexpressing Notch4 in M231shN4 cells suppressed clone formation. *P < 0.05.

Since low Notch4 expression is seemingly associated with TNBC recurrence with lymph node metastasis based on the Kaplan–Meier plotter, we explored whether the knockdown of Notch4 in the TNBC cell line promoted tumorigenesis in TNBC cells. We generated a lentivirus expressing Notch4 shRNA. The knockdown of Notch4 in TNBC MDA-MB-231 cells was confirmed using real-time RT-PCR, western blot, and promoter reporter assay (Fig. 2A–C). To confirm that Notch4 depletion promotes tumorigenesis in TNBC cells, we assessed the number of clones by clone expansion assay after 10 days’ culture. The knockdown of Notch4 in TNBC cell MDAMB231 significantly enhanced clone formation (Fig. 2D, E). Similar results were obtained in TNBC cells BT549 and Hs578T depleting Notch4 (Supplementary Fig. 2SA–SF). To further explore whether Notch4 inhibits tumorigenesis in TNBC, we overexpressed Notch4 in TNBC cell MDAMB231 with depleting Notch4. The overexpression of Notch4 was confirmed by real-time RT-PCR and western blot. As expected, Notch4 overexpression in TNBC cell MDAMB231 with depleting Notch4 suppressed clone formation by clone expansion assay after 10 days’ culture. Thus, these results strongly supported the idea that the knockdown of Notch4 promotes the tumorigenic ability of TNBC cells in vitro.

### Silencing Notch4 inhibiting metastasis in TNBC cells

**Fig. 3:**
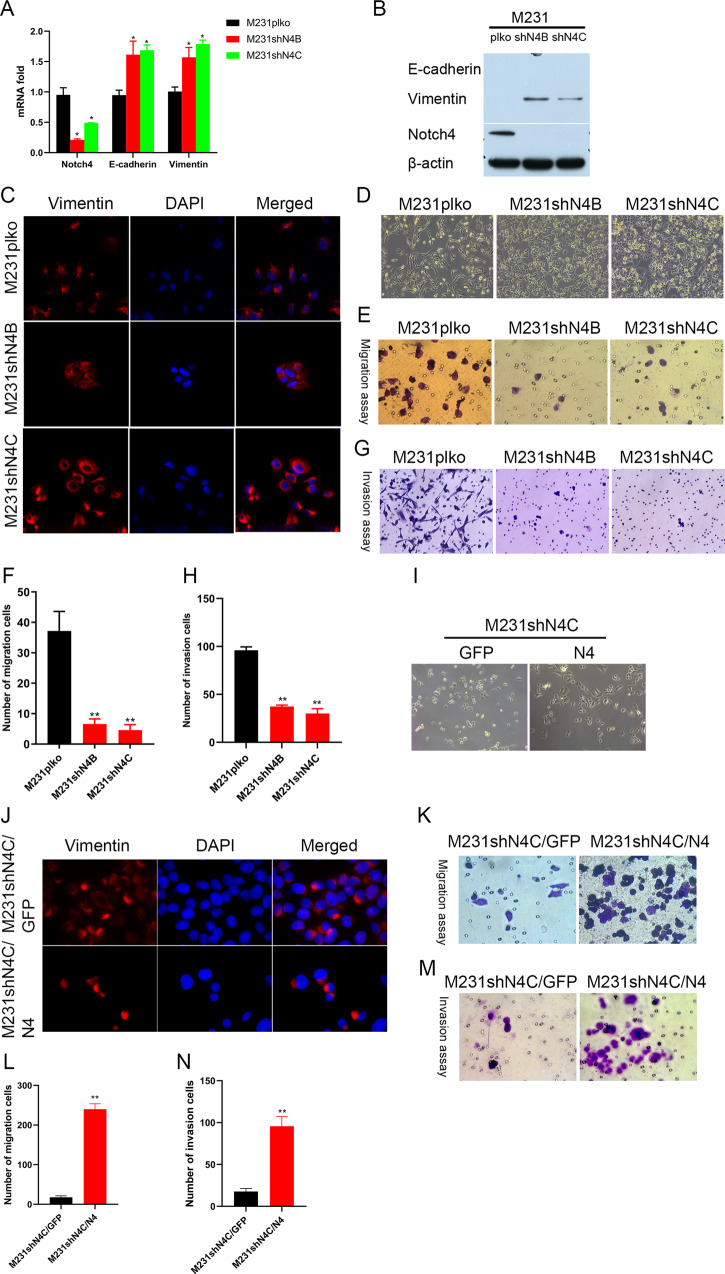
Notch4 depletion of TNBC cells suppresses migration in vitro. **A** The knockdown of Notch4 in TNBC cells enhanced E-cadherin and Vimentin expression by real-time PCR. *P < 0.05. **B** Notch4 knockdown in TNBC cells enhanced Vimentin expression by West blot analysis. **C** Notch4 depletion in TNBC cells affects Vimentin location by immunofluorescence staining. **D** Knocking down Notch4 in TNBC cells affects cellular morphology. **E**, **F** Notch4 depletion in TNBC cells suppressed migration assessed by migration assay. **P < 0.01. **G**, **H** The knockdown of Notch4 in TNBC cells inhibited invasion assessed by invasion assay. **P < 0.01. **I** Overexpressing Notch4 in M231shN4 cells rescued cellular morphology alteration. **J** Notch4 overexpression in M231shN4 cells rescued the expression of Vimentin location. **K**, **L** Overexpressing Notch4 enhanced migration of M231shN4 cells assessed by migration assay. *P < 0.01. **M**, **N** Notch4 overexpression promoted the invasion of M231shN4 cells assessed by invasion assay. **P < 0.01.

Since the knockdown of Notch4 promoted clone formation efficiency in TNBC cells and tumorigenicity was linked to epithelial-mesenchymal transition (EMT) and metastasis [29, 30], we explored whether Notch4 knockdown played a role in EMT and metastasis in TNBC cells. Partial EMT cells were found to be required for metastasis formation and cancer progression [31, 32]. As expected, the knockdown of Notch4 expression in TNBC cells enhanced Vimentin expression, a mesenchymal status marker, and E-cadherin expression, an epithelial status marker, indicating that depletion of Notch4 expression in TNBC cells boosted breast cancer metastasis (Fig. 3A, B). However, the knockdown of Notch4 shifted the spindle-shaped morphology to cobblestone-like morphology, suggesting that Notch4 depletion inhibited breast cancer metastasis (Fig. 3D). Indeed, migration assay and invasion assay confirmed that silencing Notch4 inhibited migration and invasion of TNBC cells (Fig. 3E–H). Similar results were obtained in TNBC cells BT549 and Hs578T depleting Notch4 (Supplementary Fig. 3SA–SD). Vimentin is at the core of epithelial-mesenchymal transition and metastasis in cancer cells [33, 34]. In order to explore the paradoxical outcome of Notch4 knockdown on migration, we detected the location of Vimentin expression in Notch4-depleted TNBC cells. Surprisingly, the depletion of Notch4 in TNBC cells shifted the location of Vimentin location from clustered on one side by the nucleus to distributed diffusely around the nucleus, implying that Notch4 knockdown in TNBC cells rendered Vimentin location around the nucleus uniformly and inhibited metastasis of breast cancer (Fig. 3C). In order to test the influence of Notch4 on metastasis, we overexpressed Notch4 in TNBC M231shN4 cells. As expected, overexpression of Notch4 in M231shN4C shifted cobblestone-like morphology to the slightly spindle-shaped morphology, suggesting that Notch4 enhanced breast cancer metastasis (Fig. 3I). Consistently, overexpression of Notch4 promoted migration and invasion of TNBC M231shN4 cells in vitro as assessed by migration assay and invasion assay (Fig. 3K–N). Moreover, overexpressing Notch4 shifted the location of Vimentin expression from uniformly around the nucleus to clustered on one side by the nucleus in part of TNBC M231shN4 cells (Fig. 3J). Collectively, these results indicate that silencing Notch4 in TNBC cells suppresses breast cancer migration in vitro via the location of Vimentin expression.

### Silencing Notch4 upregulates Nanog expression to promote tumorigenesis of TNBC cells through Hes3

**Fig. 4:**
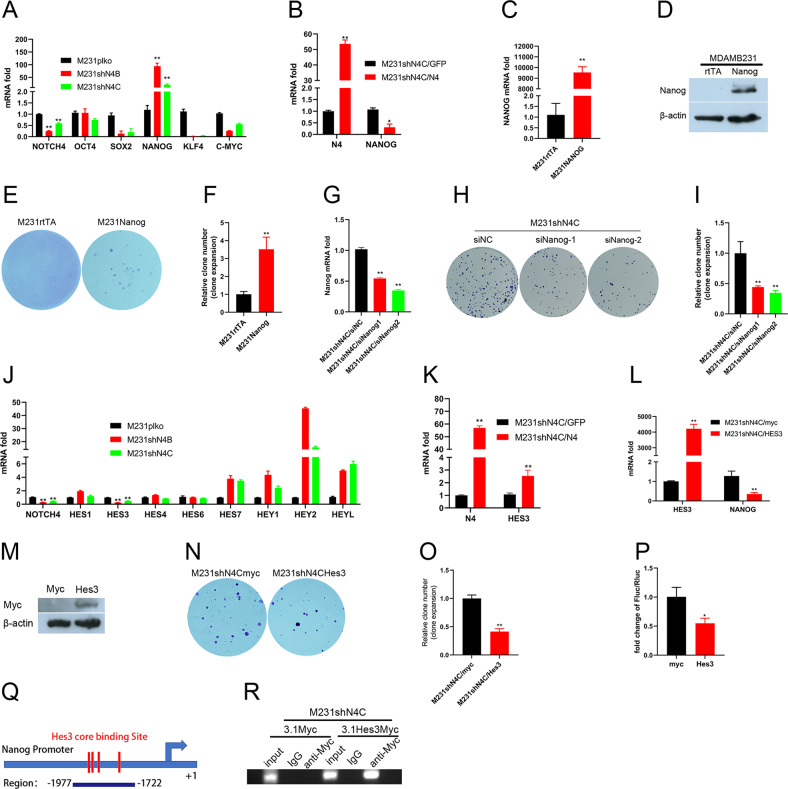
Silencing Notch4 upregulates Nanog expression to promote tumorigenesis of TNBC cells through Hes3. **A** Silencing Notch4 in TNBC cells affected mRNA expression of pluripotency factors by real-time PCR. **P < 0.01. **B** Overexpression of Notch4 in M231shN4C cells inhibited Nanog expression by real-time PCR. *P < 0.05, **P < 0.01. **C** Overexpression of NANOG in MDA-MB-231 cells was confirmed by real-time PCR. **P < 0.01. **D** Overexpression of Nanog in MDA-MB-231 cells was confirmed by western blot analysis. **E**, **F** Overexpression of Nanog in MDA-MB-231 cells enhanced clone formation. **P < 0.01. **G** The knockdown of Nanog in M231shN4C using siRNA was confirmed by real-time PCR. **P < 0.01. **H**, **I** Depletion of Nanog expression in M231/shN4C cells reduced clone formation. *P < 0.01. **J** Silencing Notch4 in TNBC cells affected mRNA expression of downstream target genes by real-time PCR. **P < 0.01. **K** Overexpression of Notch4 in M231shN4C cells upregulated HES3 expression by real-time PCR. **P < 0.01. **L** Overexpression of HES3 in M231shN4C cells inhibited Nanog expression by real-time PCR. **P < 0.01. **M** Overexpression of Hes3/myc in M231shN4C cells was confirmed using myc tag antibody by western blot analysis. **N**, **O** Overexpression of Hes3 in M231shN4C cells reduced clone formation. **P < 0.01. **P** Overexpression of HES3 in M231shN4C cells suppressed NANOG promoter activity by promoter reporter assay. Promoter activity was expressed as the ratio of firefly luciferase/Renilla luciferase. *P < 0.05. **Q**, **R** Hes3 bound to NANOG promoter by ChIP analysis. ChIP assay was done in M231shN4C cells transfected with control myc or fusion gene HES3-myc and NANOG promoter was ChIP-ed with anti-myc or IgG control.

As we know, accumulating evidence has demonstrated that TNBC cells harbor the highest percentage of cancer stem cells [35, 36]. Previously, Notch pathway activation was found to be not required in undifferentiated human embryonic stem cells (hESCs) but required for hESCs to initiate all three embryonic germ layers differentiation. Blockade of Notch activation repressed expression of molecular markers of three embryonic germ layers differentiation but strongly promoted expression of pluripotency transcription factors like Nanog [37]. Since the depletion of Notch4 promoted the tumorigenic ability of TNBC cells, we inferred that the pluripotency factors may play an important role in promoting the tumorigenic ability of TNBC cells depleting Notch4 [38, 39]. Interestingly, real-time PCR revealed that the depletion of Notch4 in TNBC cells did not affect pluripotency factor OCT4 expression or reduce the expression of pluripotency factors SOX2, KLF4, or C-myc, but strongly enhanced pluripotency factor NANOG expression (Fig. 4A). To further examine the regulation of Nanog by Notch4, we overexpressed Notch4 in TNBC cell M231shN4C. As expected, real-time PCR displayed that the overexpression of Notch4 in TNBC cells suppressed Nanog expression at the mRNA level (Fig. 4B). Previously, the expression of Nanog was found to be implicated in breast cancer tumorigenesis [20, 21]. To examine whether Nanog played an important role in the tumorigenesis of TNBC cells, we generated lentivirus overexpressing Nanog. The overexpression of Nanog in TNBC cells MDA-MB-231 was confirmed by real-time PCR and western blot analysis (Fig. 4C, D). To verify that overexpressing Nanog promotes tumorigenesis in TNBC cells, a clone expansion assay was utilized to assess the clone formation efficiency. After 10 days of culture, we found that overexpressing Nanog in TNBC cell MDA-MB-231 significantly increased clone formation ability (Fig. 4E, F). To further examine the role of Nanog in the tumorigenesis of TNBC cells, we knocked down Nanog expression by siRNA in TNBC cells M231/shN4C (Fig. 4G). As expected, the knockdown of Nanog expression in TNBC cells M231/shN4C significantly reduced the clone formation efficiency (Fig. 4H, I). Collectively, these results demonstrate that upregulation of Nanog expression mediated tumorigenesis of Notch4 depletion in TNBC cells.

HES and HEY genes encode a large family of basic helix-loop-helix repressor genes that are Notch protein effectors and play central roles in maintaining progenitor cells in an undifferentiated state [40, 41]. Real-time PCR revealed that silencing Notch4 in TNBC cells strongly repressed the expression of HES3 gene (Fig. 4J). We did not detect the expression of HES2 or HES5 genes both in Notch4 depletion or control TNBC cells. To further examine the regulation of HES3 gene expression by Notch4, we overexpressed Notch4 in TNBC M231/shN4C cells. Real-time PCR revealed that the restoration of Notch4 in M231/shN4C cells rescued HES3 gene downregulation in M231/shN4C cells (Fig. 4K). To further explore the role of Hes3 in the tumorigenesis of TNBC cells, we overexpressed the HES3 gene in TNBC M231shN4C cells (Fig. 4L, M). As expected, HES3 overexpression in M231shN4C cells boosted clone formation by clone expansion assay after 10 days of culture (Fig. 4N, O). To verify the regulation of Nanog by Hes3 in TNBC cells, we examined whether Hes3 overexpression in M231shN4C cells regulated Nanog expression by real-time PCR and promoter reporter assay. Real-time PCR revealed that Hes3 overexpression in M231shN4C cells repressed the expression of the Nanog gene in TNBC cells (Fig. 4L). Promoter reporter assay showed that HES3 overexpression in M231shN4C cells decreased Firefly luciferase expression, indicating that Hes3 inhibited the expression of Nanog in TNBC cells (Fig. 4P). Since Nanog expression was significantly regulated by Hes3, we examined whether Hes3 directly regulated Nanog expression using ChIP assay. As expected, Hes3 levels on the NANOG promoter were significantly increased in M231shN4C/HES3 cells compared with shN4C/myc cells (Fig. 4Q, R). Taken together, these results suggest that Hes3 mediated Notch4 regulating Nanog expression and tumorigenesis in TNBC cells.

### Silencing Notch4 controls Vimentin location to suppress metastasis of TNBC cells through Cdc42

**Fig. 5:**
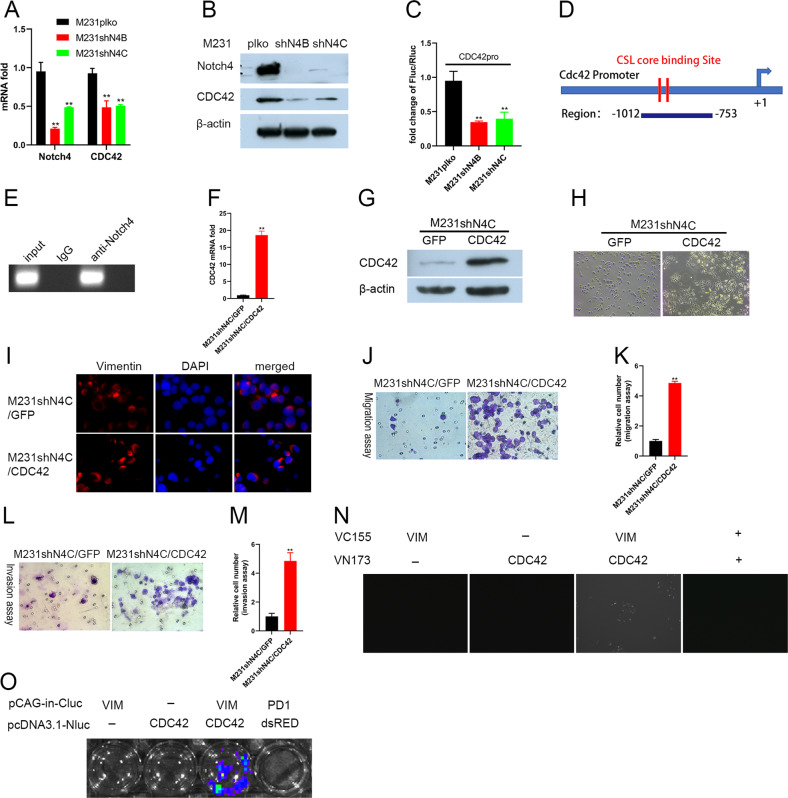
Silencing Notch4 controls Vimentin location to suppress metastasis of TNBC cells through Cdc42. **A** The knockdown of Notch4 in TNBC cells inhibited Cdc42 expression by real-time PCR. **P < 0.01. **B** The knockdown of Notch4 inhibited Cdc42 expression in TNBC cells by western blot analysis. **C** Notch4 depletion in TNBC cells suppressed Cdc42 promoter activity by promoter reporter assay. Promoter activity was expressed as the ratio of firefly luciferase/Renilla luciferase. **P < 0.01. **D**, **E** Notch4 bound to Cdc42 promoter by ChIP analysis. ChIP assay was done in MDA-MB-231 cells, and the Cdc42 promoter was ChIP-ed with anti-Notch4 or IgG control. **F** Overexpression of Cdc42 in M231shN4C cells by real-time PCR. **P < 0.01. **G** Overexpression of Cdc42 in M231shN4C cells by western blot analysis. **H** Overexpression of Cdc42 in M231shN4C cells changed the morphology from a cobblestone-like one to a slightly spindle-shaped one. **I** Overexpression of Cdc42 in M231shN4C cells altered the location of Vimentin expression. **J**, **K** Overexpression of Cdc42 in M231shN4C cells suppressed migration assessed by migration assay. **P < 0.01. L, M Overexpression of Cdc42 in M231shN4C cells inhibited invasion assessed by invasion assay.. **P < 0.01. **M** Overexpression of Cdc42 in M231shN4C cells altered the location of Vimentin expression. **N** The binding of Cdc42 to Vimentin was confirmed by the Bimolecular Fluorescence Complementation assay. **O** The binding of Cdc42 to Vimentin was confirmed by Firefly Luciferase Complementation Imaging Assay.

Previously research showed that cell polarity is essential for effective cell mobility, and Cdc42 plays a central role in cell polarity establishment by recruiting its effectors to form a crescent-shaped polar cap, which orchestrates rearrangements of the actin cytoskeleton [42–44]. They found that the polar cap number increases with Cdc42 cytoplasmic concentration, and deposition of Cdc42 to the cap further stimulates actin accumulation at this site. Therefore, we inferred that silencing Notch4 in TNBC cells may cause a decrease of polar cap via downregulating Cdc42 cytoplasmic concentration and inhibits cell mobility. Expectedly, the knockdown of Notch4 in TNBC cells significantly reduced Cdc42 expression at the mRNA level and protein level (Fig. 5A, B). To further explore the molecular mechanisms underlying the regulation of Cdc42 expression by Notch4, a CDC42 promoter vector was created, and promoter reporter assays were performed. The results confirmed that the depletion of Notch4 in TNBC cells suppressed CDC42 promoter activity, indicating that silencing Notch4 in TNBC cells reduced Cdc42 expression (Fig. 5C). In order to examine whether Notch4 regulates Cdc42 expression in TNBC cells, we performed ChIP assay to assess the binding of Notch4 at CDC42 gene promoter. Indeed, we found that Notch4 is bound to the promoter region of the CDC42 gene (Fig. 5D, E). Taken together, these results indicate that Notch4 regulates Cdc42 expression in TNBC cells.

To examine whether Cdc42 played an important role in the migration of TNBC cells, we overexpressed Cdc42 in M231shN4C cells. The overexpression of Cdc42 in M231shN4C cells was confirmed by real-time PCR and western blot analysis (Fig. 5F, G). Interestingly, Cdc42 overexpression in M231shN4C cells changed the cobblestone-like morphology to the slightly spindle-shaped morphology, indicating that Cdc42 overexpression in M231shN4C cells enhanced the mobility of breast cancer (Fig. 5H). As expected, Cdc42 overexpression increased migration and invasion of TNBC M231shN4 cells in vitro as assessed by migration assay and invasion assay (Fig. 5I–L). Moreover, Cdc42 overexpression altered the location of Vimentin expression from uniformly around the nucleus to clustered on one side by the nucleus in part of TNBC M231shN4 cells (Fig. 5M). The role of Vimentin was found to stabilize microtubule network organization and enhance cell polarity in directed cell migration. The location of Vimentin in the cells is oriented in the direction of migration at the wound edge. The knockdown of Vimentin causes a defect of polarity and reduces migrating speed [45]. Vimentin fibers were aligned with fibroblast branching and migration direction in fibroblasts and required for the anisotropic orientation of traction stresses, indicating that vimentin’s role in cell motility is to govern the alignment of traction stresses that permit single-cell migration [46]. Since Cdc42 and Vimentin are all implicated in the formation of crescent-shaped polar cap of cell polarity and migration, we proposed that Cdc42 may bind to Vimentin and govern the location of Vimentin expression in TNBC cells. To test this possibility, we utilized the Bimolecular Fluorescence Complementation assay and Firefly Luciferase Complementation Imaging Assay to detect Cdc42-Vimentin interaction [47]. The fusion proteins at a low level were utilized in the Bimolecular Fluorescence Complementation assay to avoid self-assembly and remove the generation of false-positive signals. In each sample, 10 ng vector is the appropriate quantity to remove the false positive without affecting specific signals [48]. Indeed, in the Bimolecular Fluorescence Complementation assay, we observed the Venus fluorescence in the group, including Cdc42 fusion protein and Vimentin fusion protein, under the fluorescence microscope, indicating that the binding of Cdc42 to Vimentin occurred. As a control, we did not observe the Venus fluorescence in the group including control vectors VN173 and VC155, indicating that 10 ng split fluorescent protein fragments did not self-assemble by random collision and generate false-positive signals (Fig. 5N). Similarly, in the Firefly Luciferase Complementation Imaging Assay, we detected Bioluminescent imaging in the group transfecting with Cdc42 fusion protein and Vimentin fusion protein by IVIS Lumina III imaging system, confirming the binding of Cdc42 protein to Vimentin. We also did not detect Bioluminescent imaging in the control group transfecting with 10 ng control vector PCAG-IN-PD1-CLuc and 10 ng control vector pcDNA3.1-NLuc-dsRED for 3 days (Fig. 5O). Taken together, our results suggest that Cdc42 protein mediated the function of silencing Notch4 in TNBC cells that inhibits Vimentin cluster formation and metastasis.

## Discussion

The Notch signaling cascade is an evolutionarily conserved pathway that plays a key role in cell fate decision during development [18]. Disorder of Notch signal pathway regulation can result in cancer occurrence and metastasis [49]. Evidence suggests that four Notch proteins have distinct activities and outcomes in tumorigenesis [50]. Previous research showed that Notch4 played a role in maintaining quiescent mesenchymal-like breast cancer stem cells and promoting epithelial-mesenchymal transition in TNBC cells, indicating that Notch4 is a potential therapeutic target for TNBC [14, 15]. In our study, we found that silencing Notch4 inhibited migration and invasion ability but promoted tumorigenic ability in TNBC cells. Interestingly, we found that silencing Notch4 upregulated pluripotency factor Nanog expression to enhance tumorigenesis in TNBC cells. More importantly, silencing Notch4 affected Vimentin distribution but not Vimentin expression to inhibit migration via Cdc42 expression in TNBC cells. Therefore, our study suggests that silencing Notch4 in TNBC cells inhibits migration ability and enhances tumorigenesis ability via Cdc42 and Nanog, respectively.

Previous research showed that high expression of Nanog was a poorer prognostic factor in the TNBC subtype [51]. GFP reporter to sort Nanog^+^ cells from TNBC cells significantly increased cancer stem cell activity in vitro and tumorigenic ability in vivo [52]. Cx26 complexed with the pluripotency transcription factor NANOG and focal adhesion kinase (FAK) promotes cancer stem cell self-renewal of TNBC cells but not luminal breast cancer cells [53]. These studies indicated that Nanog plays an important role in cancer stem cell activity in TNBC cells. Our results showed that silencing Notch4 upregulated Nanog expression and enhanced the tumorigenic ability of TNBC cells, consistent with previous findings of Nanog controlling cancer stem cell activity in TNBC cells.

In the gland-like structures formed by human renal cell carcinoma cells, the intracellular localization of Vimentin is most concentrated at the basal pole, indicating cell polarity has a close relationship with the subcellular localization of Vimentin [54]. Vimentin fibers were aligned with fibroblast branching and migration direction, suggesting that the role of Vimentin in cell motility is to control the alignment of traction stresses that permit single-cell migration [46]. Vimentin interacting with microtubules contributes to polarity maintenance in migrating cells. Knockdown of Vimentin prevents cell polarization and migration properly during wound healing, indicating the role of Vimentin in maintaining cell polarization in general and migration in particular [45, 55]. These studies implied that Vimentin plays an important role in cell polarity formation and migration. Our results showed that silencing Notch4 in TNBC cells rendered Vimentin expression from concentrated distribution and bell-shaped location on one side of the nucleus to diffuse distribution around the nucleus. Rescued assay showed that overexpression of Notch4 in M231shN4C cells caused Vimentin expression from diffuse distributed around the nucleus to concentrated distribution and bell-shaped location on one side of the nucleus again. Combined with previous research, we inferred that concentrated distribution of intracellular Vimentin on one side of the nucleus induced cell polarity formation and possibly drag nucleus mobility that promotes cell migration, and silencing Notch4 results in diffuse distribution of intracellular Vimentin around the nucleus and inhibits cell migration in TNBC cells. In order to elucidate the molecular mechanisms of Vimentin distributing concentrated on one side of the nucleus and regulating cell polarity formation, Cdc42 was found to be at the center of cell polarity formation in previous research [56]. Cdc42 protein was localized and clustered in the cytoplasm and promotes cell polarity formation, which is essential for cell motility and migration [42, 57, 58]. Our results showed that silencing Notch4 reduced Cdc42 expression and rendered diffuse distribution of Vimentin expression in TNBC cells. Rescued assay showed that overexpression of Notch4 upregulated Cdc42 expression and caused Vimentin localization in a bell shape on one side of the nucleus in M231shN4C cells. More importantly, Cdc42 overexpression in TNBC M231shN4 cells altered Vimentin expression from diffuse distribution around the nucleus to a concentrated location on one side by the nucleus, indicating that Cdc42 mediates Notch4 regulating Vimentin localization and polarity formation in TNBC cells. Considering that Cdc42 binds to the microtube to foster polarity establishment and the binding of Vimentin to the microtube, we inferred that Cdc42 possibly binds to Vimentin to promote the concentrated distribution of Vimentin location and polarity formation [45, 59]. As expected, the Bimolecular Fluorescence Complementation assay and Firefly Luciferase Complementation Imaging Assay confirmed that Cdc42 is bound to Vimentin in TNBC cells. Taken together, our results suggest that silencing Notch4 in TNBC cells decreased Cdc42 expression and rendered diffuse distribution around the nucleus of Vimentin expression, thereby leading to cell polarity disappearance and migration inhibition.

Increasing evidence proved that the Notch signal pathway plays a vital role in tumorigenesis and the progression of breast cancer. Therapeutic strategy for targeting Notch signal has been studied for decades, but clinical outcome fails to meet the expectation. GSIs, like AL101, PF-03084014, and RO4929097, have been developed as a treatment for triple-negative breast cancer in phase II clinical trials, but most of these studies were terminated since the serious adverse events and poorer outcomes [60]. The reasons for adverse events of GSIs may be due to the inhibition of all Notch proteins. Thereby, clarity of activity and function of each individual Notch protein in a subtype of breast cancer facilitates the development of Notch-targeting therapeutic agents with safety and efficacy. Previous research showed that the knockdown of Notch4 in TNBCS suppressed cancer stem cell activity and migration, indicating that Notch4 is a potential target for tumor intervention [14]. Our results showed that silencing Notch4 suppressed migration but enhanced tumorigenic ability in TNBC cells, suggesting that Notch4 should not be a potential therapeutic target for TNBC.

## Conclusion

**Fig. 6:**
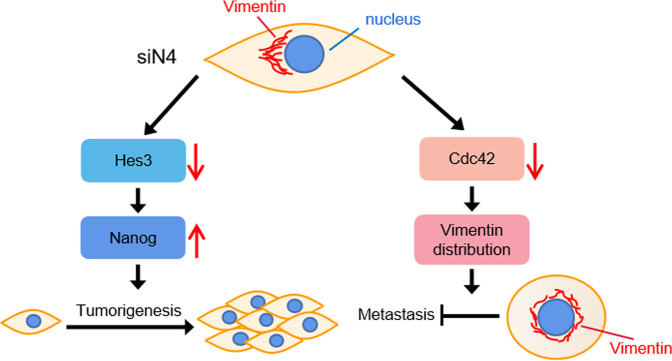
Proposed model of Notch4 knockdown affecting tumorigenesis and metastasis of TNBC cells. Silencing Notch4 in TNBC cells enhances Nanog expression via Hes3 to promote tumorigenesis and affects Vimentin distribution via Cdc42 expression to inhibit metastasis.

In conclusion, our study demonstrated that silencing Notch4 in TNBC cells suppressed metastasis ability but promoted tumorigenic ability. Mechanistically, silencing Notch4 upregulated Nanog expression, a transcription factor required for maintaining the pluripotency of embryonic stem cells, via Hes3 to promote tumorigenesis. Unexpectedly, silencing Notch4 rendered diffuse distribution around the nucleus of Vimentin but did not decrease Vimentin expression to suppress metastasis in TNBC cells. Generally, upregulation of Vimentin expression and downregulation of E-cadherin expression are regarded as the key molecular events for EMT shift and metastasis. Notably, Cdc42, a key molecular for polarity formation and metastasis mediates Notch4 regulating Vimentin distribution (Fig. 6). Therefore, our demonstration indicated Notch4 will not be a potential therapeutic target for TNBC.

## Supplementary information

Supplemental Material clean version

Full and uncropped western blots
