# Supplementary material for: Silencing Notch4 promotes tumorigenesis and inhibits metastasis of triple-negative breast cancer via Nanog and Cdc42
Source: Cell Death Discov. 2023 May 6;9:148. doi: 10.1038/s41420-023-01450-w (PMC10164131; doi:10.1038/s41420-023-01450-w)
Supplement: Supplementary file 1 — Supplemental Material clean version [file 41420_2023_1450_MOESM1_ESM.docx]

**Silencing Notch4 promotes tumorigenesis and inhibits** **metastasis of triple-negative breast cancer via Nanog and Cdc42**

Yuan Tian^#1,2^, Peipei Zhang^#1,2^, Yajun Mou^1,2^, Wenxiu Yang^1^, Junhong Zhang^1^, Qing Li^3^, Xiaowei Dou^*2^

^1^Department of Pathology, ^2^Clinical Research Center, ^3^Department of Orthopedics, the Affiliated Hospital of Guizhou Medical University, Guiyang 550004, Guizhou, China.

# Y. Tian and P. Zhang contributed this work equally.

*Correspondence author Xiaowei Dou, ([douxw@gmc.edu.cn](mailto:douxw@gmc.edu.cn))


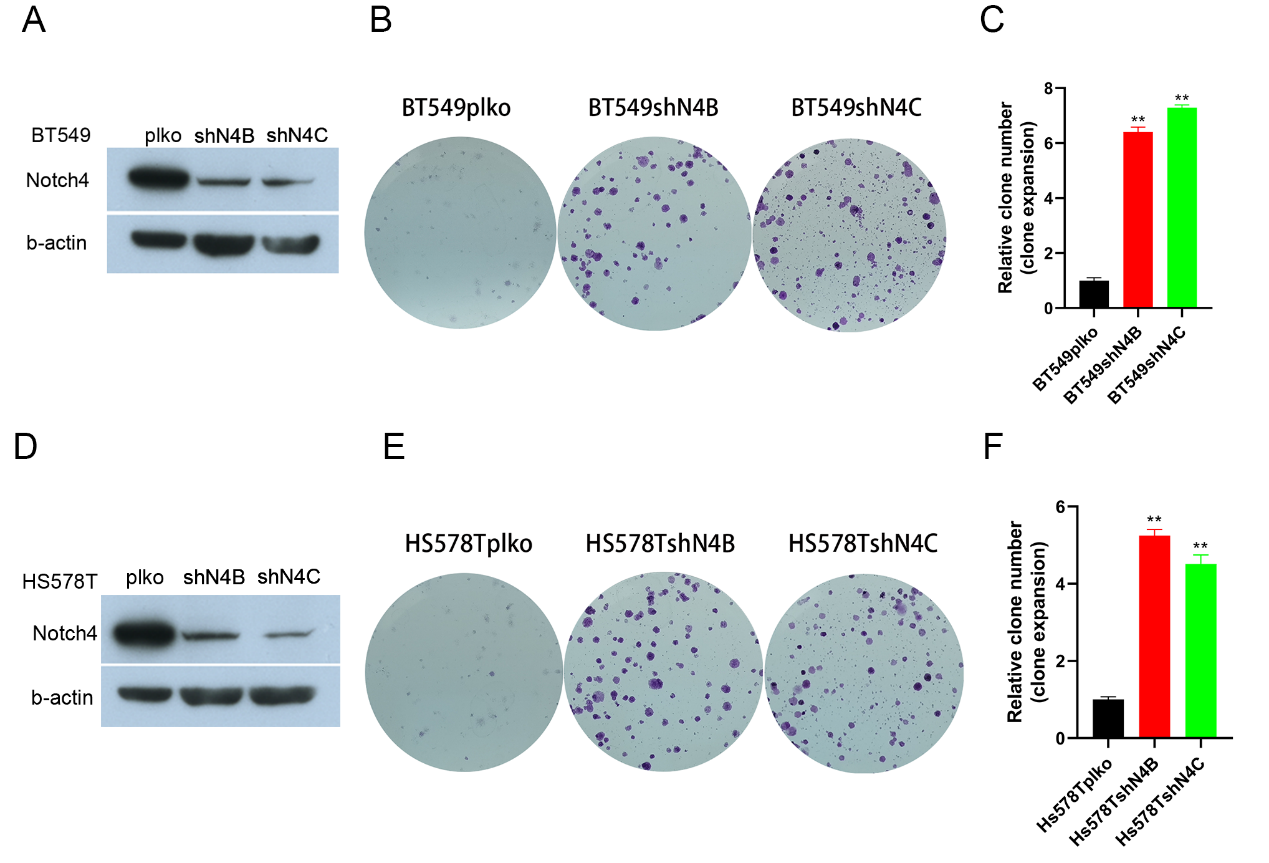


**Figure 2S The knockdown of Notch4 of TNBC cells promotes tumorigenesis in vitro.**

(A) Notch4 knockdown in BT-549 cells was confirmed by Western blot analysis.

(B, C) Notch4 knockdown of BT-549 cells increased clone formation. *P< 0.05.

(D) Silencing Notch4 in Hs 578T cells by Western blot analysis.

(E, F) Silencing Notch4 in Hs 578T cells increased clone formation. *P< 0.05.


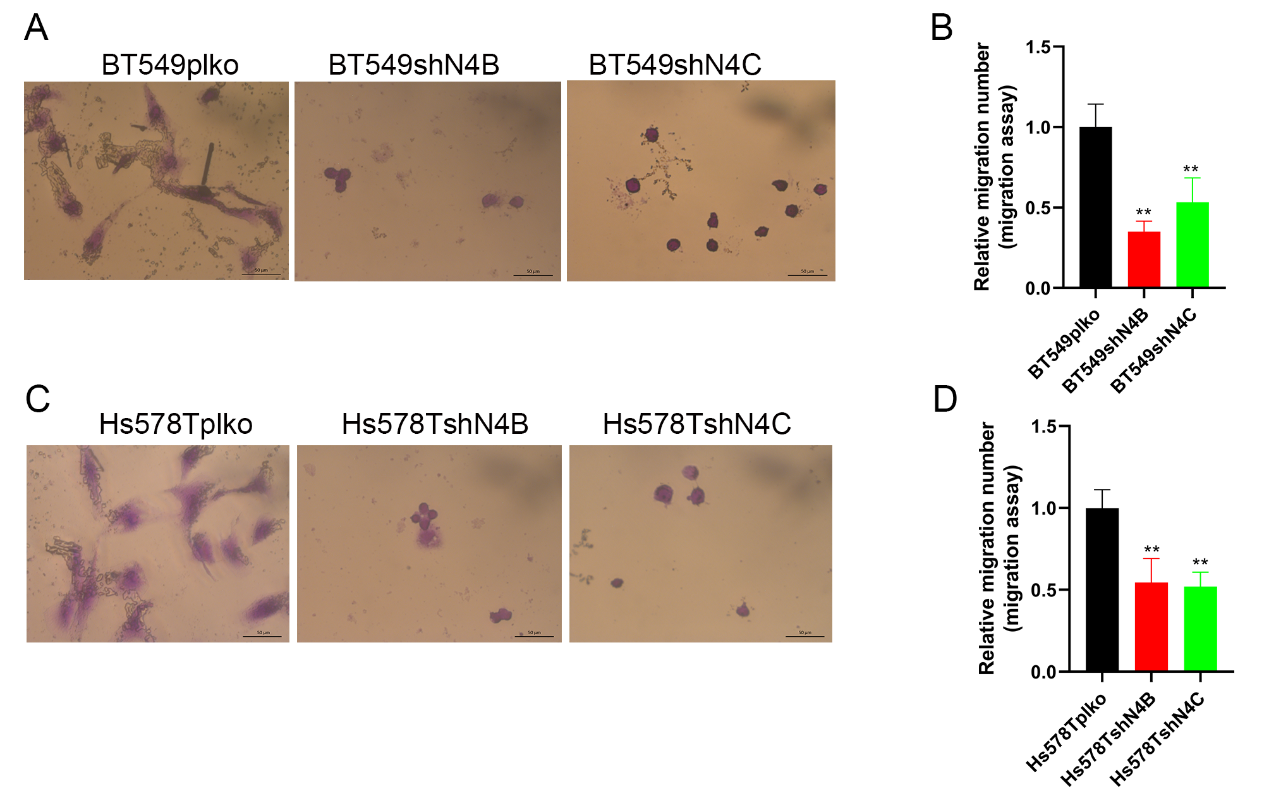


**Figure. 3S Notch4 depletion of TNBC cells suppresses migration in vitro.**

(A, B) Notch4 depletion in BT-549 cells suppressed migration assessed by migration assay. *P< 0.01.

(C, D) The knockdown of Notch4 in Hs 578T cells inhibited invasion assessed by invasion assay. *P< 0.01.

**Materials and methods**

**Cells, vectors and reagents**

Breast cancer cell lines MDA-MB-231, Hs 578T and BT-549 were ordered from Cell Bank, Type Culture Collection in Chinese Academy of Science (CBTCCCAS, Shanghai, China). Lentivirus vectors including control vector Plko.1, silecing Notch4 vectors shN4B and shN4C were ordered from Sigma-Aldrich. The siRNA sequence are: shN4B: cgataaagatgcccaggacaa; shN4C: ctgcgataatgcgaggaagat. Three copies of Hes1 promoter (-45bp~-90bp) containing CSL binding elements was cloned into the enzyme site MluI and XhoI of pGL3-basic as Notch responsive element (Promega). Control vector pRL-SV40 was from Promega. GFP (fused with 3×flag/6×his tag), Notch4ICD (fused with mCherry) or Cdc42 (fused with 3×flag/6×his tag) were subcloned into the enzyme site BamHI and AscI of pcDNA3.1-Hygro vector (Life Technologies). rtTA as control or Nanog were subcloned into the enzyme site EcoRI and NotI of pCDH-CMV-MCS-EF1-CopGFP-T2A-Puro vector (SBI). siRNAs sequence was as: siNC: TTCTCCGAACGTGTCACGTTT, siNanog-1: AAGGGTTAAGCTGTAACATAC; siNang-2: GCAACCAGACCTGGAACAATT (GenePharma, China). Hes3 was subcloned into the enzyme site BamHI and AscI of pcDNA3.1/myc-His A vector (Invitrogen). NANOG promoter (-1977~-1722/-100bp~+50bp) was cloned into the enzyme site KpnI and HindIII of pGL4.17[Luc2Neo] (Promega). CDC42 promoter (-960~-640/-150bp~+50bp) was cloned into the enzyme site KpnI and HindIII of pGL4.17[Luc2Neo] (Promega). VIMENTIN CDS was subcloned into the enzyme site EcoRI and KpnI of pBiFC-VC155 vector. CDC42 CDS was subcloned into the enzyme site EcoRI and KpnI of pBiFC-VN173 vector. VIMENTIN CDS or PD1 were subcloned into the enzyme site NotI and AscI of pCAG-IN-CLuc vector. CDC42 CDS or dsRED CDS were subcloned into the enzyme site BamHI and AscI of pcDNA3.1-NLuc-hygro (Life Technologies)^1^. The primary antibodies anti-Notch4 (L5C5, CST), anti-ERa (D8H8, CST), anti-Nanog (D73G4, CST), anti-Cdc42 (11A11, CST), anti-E-cadherin (24E10, CST), anti-Vimentin (D21H3, CST), anti-myc (9E10, Sigma), anti-β-actin (C4, Santa), goat against rabbit IgG-HRP (sc-2357, Santa) and m-IgGκBP-HRP (sc-516102, Santa) were ordered. The lentivirus was synthesized by control vector Plko.1, silecing Notch4 vectors shN4B or shN4C with package vectors psPAX2 and pMD2.G in 293T cells using Lipofectamine 2000 (Invitrogen). The lentivirus was filtered through 0.45 uM filter and used to infection. MDA-MB-231 cells was infected with the control lentivirus Plko.1 and next day selected by 0.5 ug/ml puromycin (AMRESCO) for 2 days. The cell assigned as M231plko and so on.

The control rtTA lentivirus or overexpressing Nanog lentivirus were synthesized and infected the cells describled as above. MDA-MB-231 cells infected with rtTA lentivirus or overexpressing Nanog lentivirus were assigned as M231rtTA and M231Nanog.

**Western blot**

Western blot was done as describled previously^2^. In brief, cells were washed with cold PBS, lysed with RIPA buffer, separated by 12% SDS-PAGE gel and transferred onto PVDF membranes. The membranes were immunoblotted with primary antibodies against anti-Notch4 (1:1000), anti-ERa (1:1000), anti-β-actin (1:1500), anti-Nanog (1:1000), anti-Cdc42 (1:1000), anti-E-cadherin (1:1000), anti-Vimentin (1:1000), anti-myc (1:2000). The membranes were incubated with goat against rabbit IgG-HRP (1:1000) or m-IgGκBP-HRP (1:1,000) and then detected by Immobilon Western Chemiluminescent HRP Substrate (Thermo Fisher Scientific).

**Real-time PCR**

Total RNA was extracted from cells using RNAiso Plus (TaKaRa). 800 ng of total RNA was reversely transcribled to synthesize cDNA using PrimeScript RT Reagent kit (Takara) according to the manufacturer’s instruction. The real-time PCR was done using TB Green Premix Ex Taq II kit (TaKaRa) and the Vii 7 Real-time PCR Detection system (Bio-Rad). Each sample is done in triplicate. The primers were as follows: **β-actin** forward, 5’- TGACGTGGACATCCGCAAAG -3’ and reverse, 5’- CTGGAAGGTGGACAGCGAGG -3’; **Notch4** forward, 5’-CGAGGAAGATACGGAGTGGC -3’ and reverse, 5’- GGATCGGAATGTTGGAGGCA’; **E-cadherin** forward, 5’-AAAGGCCCATTTCCTAAAAACCT-3’ and reverse, 5’-TGCGTTCTCTATCCAGAGGCT -3’; **Vimentin** forward, 5’-GCCCTAGACGAACTGGGTC-3’ and reverse, 5’-GGCTGCAACTGCCTAATGAG-3’; **OCT4** forward, 5’-GGAGGAAGCTGACAACAATGAAA-3’ and reverse, 5’-GGCCTGCACGAGGGTTT-3’; **SOX2** forward, 5’-TGCGAGCGCTGCACAT-3’ and reverse, 5’-TCATGAGCGTCTTGGTTTTCC-3’; **Nanog** forward, 5’-ACAACTGGCCGAAGAATAGCA-3’ and reverse, 5’-GGTTCCCAGTCGGGTTCAC-3’; **KLF4** forward, 5’- CGAACCCACACAGGTGAGAA-3’ and reverse, 5’-GAGCGGGCGAATTTCCAT-3’; **C-MYC** forward, 5’-AGGGTCAAGTTGGACAGTGTCA-3’ and reverse, 5’-TGGTGCATTTTCGGTTGTTG-3’; **Hes1** forward, 5’- CACTGATTTTGGATGCTCTGAAG-3’ and reverse, 5’-CACTTGGGTCTGTGCTCAG-3’; **Hes2** forward, 5’-TCGGTTTCCCTTTGCGTG-3’ and reverse, 5’- AGGCTCTGGTTGATGCG-3’; **Hes3** forward, 5’-GCAAATTGGAGAAGGCCGAC-3’ and reverse, 5’-TGCAAGGAGTTCTGAAGGCT-3’; **Hes4** forward, 5’-GGACGCCCTCAGAAAAGAG-3’ and reverse, 5’-GGTACTTGCCCAGAACGG-3’; **Hes5** forward, 5’-CAGCCCCAAAGAGAAAAACC-3’ and reverse, 5’-CGGCCTTCTCCAGCTTG-3’; **Hes6** forward, 5’-AGGATGAGGACGGCTGG-3’ and reverse, 5’-CACTTCGGCGTTCTCCAG-3’; **Hes7** forward, 5’-GAGCTGAGAATAGGGACGG-3’ and reverse, 5’-TTTCTCCAGCTTCGGGTTC-3’; **Hey1** forward, 5’-TTGAGAAGCAGGTAATGGAGC-3’ and reverse, 5’-CTCCGATAGTCCATAGCAAGG-3’; **Hey2** forward, 5’-GACAGTGGATCATTTGAAGATGC-3’ and reverse, 5’-CTGTTAGGCACTCTCGGAATC-3’; **HeyL** forward, 5’-CATCGACGTGGGCCAAG-3’ and reverse, 5’-CGTTTCTCTATGATCCCTCTGC-3’; **Cdc42** forward, 5’-TGGAGTGTTCTGCACTTACACA-3’ and reverse, 5’- GCAGCCAATATTGCTTCGTCA -3’.

**Luciferase assay.**

Cdc42 promoter or Notch responsive promoter reporter assay was done using MDA-MB-231 cells with silencing Notch4 or control. Nanog promoter reporter assay was done using 293T cells transfected with Hes3 or control vector. One day before transfection, 1*10^4^ cells were seeded in each well of 96 well plate. The cells were co-transfected with 100 ng reporter construct, 10 ng internal control vector pRL-SV40 and/or 140 ng transfection vector in each well of 96-well plate using transfection reagent Lipofectamine 2000 (Thermo Fisher) according to the manufacturer’s protocol. Each is done in triplicate. After transfectin 24 hours , luciferase activities were measured using the Dual-Luciferase Reporter assay (Promega) according to the manufacturer’s protocol and Synergy H4 Hybrid Microplate detection system (BioTek). The promoter activity was relatively assessed by calculating the ratio of Firefly luminescence to Renilla luminescence.

**ChIP assay**

The ChIP assays were performed as depicted previously^2^. The binding of Hes3 protein to Nanog promoter was done using M231shN4C cell transfected with control myc or gene fusion Hes3-myc. The binding of Notch4 protein to Cdc42 promoter was done using MDA-MB-231 cell. Chromatin was sheared

by applying 3 sonication cycles for 10 s at 20% power and 3 s rest on ice (Bandelin Sonopuls HD 3100). Antibodies for ChIP assays were ordered from the following companies: anti-Notch4 (5 ul for each experiment, L5C5, CST), anti-myc (2 ug for each experiment, 9E10, Sigma) and mouse IgG (2 ug for each experiment, sc-2025, Santa). PCR amplification for proposed Hes3-binding Nanog promoter fragement (-2000 ~ -1681 bp) and proposed Notch4-binding Cdc42 promoter fragement (-932 ~ -753 bp) was performed using 2×Taq PCR MasterMix (KT201, Tiangen, China) according to the manufacturer’s protocol and ProFlex PCR system (Applied Biosystems). The primers used for PCR are as follows: **CDC42pro** forward, 5’-CTTTTTATTCTTTCAGAAAA-3’ and reverse, 5’-AGTACCAAAGAGTCATTAAT-3’; **NANOGpro** forward, 5’-CAGTTAATTTTTGTATTTTTA-3’ and reverse, 5’-GTGTCTAGGGTAAGAGCCTC-3’.

**Clone formation assay**

200 cells were seeded in each well of 6 well plate. Each is done in triplicate. Cell culture media were changed every days. Each is done in triplicate. After 10 days’ culture, the cells were fixed with 4% formalin for minutes and stained with 0.1% crystal violet (Singma) for 15 minutes. The clone numbers were calculated using ImageJ software.

**Immunofluorescence staining**

The cells were fixed with 4% PFA for 15 minutes and then permeabilized with 0.5% Triton X-100 for 5 minutes. After blocked with 2.5% goat serum for 1 hour, the cells were incubated with antibody anti-Vimentin (1:100, D21H3 , CST) overnight at 4˚C. Antibody binding was visualized by incubation with a Anti-Rabbit IgG Fragment (Alexa Fluor 555 Conjugate, 1:1000, #4413, CST) at room temperature for 1 hour. Anti-Rabbit IgG Fragment was used alone to stain cells as a negative control to assess non-specific binding. The cells were then stained with 0.5 ug/mL DAPI (#4083, CST) at room temperature for 3 minutes and imaged using an Zeiss Axio Imager fluorescence microscope.

**Cell migration and invasion assay**

Cell culture insert (8 μM pore size; BD) or coated with 180 ul matrigel of each insert (356234, Corning) were used to perform migration and invasion assays, respectively. After serum-starved for 24 hours, a 200 ul cell suspension containing 5000 cells and serum-free medium was plated into the upper chamber. 500 µL of media containing 10% fetal bovine serum was added to the bottom chamber of the migration plate. Each sample was done in triplicate. After 24 hours for cell migration assay or 48 hours for cell invasion assay, the cells were fixed with ice-cold methanol for 20 minutes and then stained with 0.1% crystal violet for 15 minutes. The numbers of invaded cells were counted from 5 fields in each well at 400× magnification.

**Bimolecular Fluorescence Complementation assay**

One day before transfection, 1*10^5^ 293T cells/well were cultured in 12 well plate. The cells were co-transfected with 10 ng pBiFC-VC155-Vimentin and 10 ng pBiFC-VN173-CDC42 using transfection reagent Lipofectamine 2000 (Thermo Fisher) according to the manufacturer’s protocol. The cells were transfected with 10 ng pBiFC-VC155-Vimentin alone or 10 ng pBiFC-VN173-CDC42 alone as a negative control. The cells were co-transfected with 10 ng pBiFC-VC155 and 10 ng pBiFC-VN173 to avoid false-positive signal that caused by high concentration of split fluorescence proteins and assemble in close proximity of each other^3^. After 3 days’ transfection, the fluorescence was imaged using an Zeiss Axio fluorescence microscope.

**Firefly Luciferase Complementation Imaging Assay**

1*10^5^ 293T cells were plated in each well of 12 well plate for one day before transfection. The cells 10 ng PCAG-IN-Vimentin-CLuc and 10 ng pcDNA3.1-NLuc-CDC42 were co-transfected into 293T cells using transfection reagent Lipofectamine 2000 (Thermo Fisher) according to the manufacturer’s protocol. The cells were transfected with 10 ng control vector PCAG-IN-PD1-Cluc alone and 10 ng control vector pcDNA3.1-NLuc-dsRED alone as a negative control. The cells were co-transfected with 10 ng control vector PCAG-IN-PD1-CLuc and 10 ng control vector pcDNA3.1-NLuc-dsRED to avoid false-positive signal. After 3 days’ transfection, Bioluminescence was detected using D-Luciferin (122796, Caliper) according to the manufacturer’s instruction and IVIS Lumina III imaging system.

**Statistical analysis**

All experiments were done in triplicate. Data are shown as the mean ± SEM, and were statistically analyzed using a two-sided Student’s t-test. P＜0.05 was considered statistically significant.

Reference

1 Chen, H. *et al.* Firefly luciferase complementation imaging assay for protein-protein interactions in plants. *Plant Physiol* **146**, 368-376, doi:10.1104/pp.107.111740 (2008).

2 Dou, X. W. *et al.* Notch3 Maintains Luminal Phenotype and Suppresses Tumorigenesis and Metastasis of Breast Cancer via Trans-Activating Estrogen Receptor-alpha. *Theranostics* **7**, 4041-4056, doi:10.7150/thno.19989 (2017).

3 Mao, S., Ying, Y., Ma, Z., Yang, Y. & Chen, A. K. A Background Assessable and Correctable Bimolecular Fluorescence Complementation System for Nanoscopic Single-Molecule Imaging of Intracellular Protein-Protein Interactions. *ACS Nano* **15**, 14338-14346, doi:10.1021/acsnano.1c03242 (2021).
