# Supplementary material for: Silencing Notch4 promotes tumorigenesis and inhibits metastasis of triple-negative breast cancer via Nanog and Cdc42
Source: Cell Death Discov. 2023 May 6;9:148. doi: 10.1038/s41420-023-01450-w (PMC10164131; doi:10.1038/s41420-023-01450-w)
Supplement: Supplementary file 2 — Full and uncropped western blots [file 41420_2023_1450_MOESM2_ESM.docx]

**Silencing Notch4 promotes tumorigenesis and inhibits** **metastasis of triple-negative breast cancer via Nanog and Cdc42**

Yuan Tian^#1,2^, Peipei Zhang^#1,2^, Yajun Mou^1,2^, Wenxiu Yang^1^, Junhong Zhang^1^, Qing Li^3^, Xiaowei Dou^*2^

^1^Department of Pathology, ^2^Clinical Research Center, ^3^Department of Orthopedics, the Affiliated Hospital of Guizhou Medical University, Guiyang 550004, Guizhou, China.

\# Y. Tian and P. Zhang contributed this work equally.

*Correspondence author Xiaowei Dou, ([douxw@gmc.edu.cn](mailto:douxw@gmc.edu.cn))

Western blot figures and raw material

**Figure 1A**

**Figure 2B**

**Figure 2G**

**Figure 2S-A、D**

**Figure 3B**

**Figure 4O**

**Figure 4E**

**Figure 5B**

**Figure 5H**
